# What do Retraction Notices Reveal About Institutional Investigations into Allegations Underlying Retractions?

**DOI:** 10.1007/s11948-023-00442-4

**Published:** 2023-07-04

**Authors:** Shaoxiong Brian Xu, Natalie Evans, Guangwei Hu, Lex Bouter

**Affiliations:** 1 School of Foreign Studies, Huanggang Normal University, Huanggang, China; 2 Department of English and Communication, The Hong Kong Polytechnic University, Kowloon, Hong Kong China; 3 Department of Ethics, Law, and Humanities, Amsterdam University Medical Centers, Amsterdam, The Netherlands; 4 Department of Epidemiology and Data Science, Amsterdam University Medical Centers, Amsterdam, The Netherlands; 5 Department of Philosophy, Vrije Universiteit Amsterdam, Amsterdam, The Netherlands

**Keywords:** Research misconduct, Retraction notice, Institutional investigation, Research integrity and ethics, Allegation

## Abstract

Academic journal publications may be retracted following institutional investigations that confirm allegations of research misconduct. Retraction notices can provide insight into the role institutional investigations play in the decision to retract a publication. Through a content analysis of 7,318 retraction notices published between 1927 and 2019 and indexed by the Web of Science, we found that most retraction notices (73.7%) provided no information about institutional investigations that may have led to retractions. A minority of the retraction notices (26.3%) mentioned an institutional investigation either by journal authorities (12.1%), research performing organizations (10.3%), joint institutions (1.9%), research integrity and ethics governing bodies (1.0%), third-party institutions (0.5%), unspecified institutions (0.4%), or research funding organizations (0.1%). Comparing retraction notices issued before and after the introduction of retraction guidelines by the Committee on Publication Ethics (COPE) in 2009 revealed that those published after the guidelines’ publication were more likely to report investigations by journal authorities. Comparing retraction notices from different disciplines revealed that those from social sciences and the humanities were more likely to disclose investigations by research performing organizations than those from biomedical and natural sciences. Based on these findings, we suggest that the COPE retraction guidelines in the future make it mandatory to disclose in retraction notices institutional investigations leading to retractions.

**Supplementary Information:**

The online version contains supplementary material available at 10.1007/s11948-023-00442-4.

## Introduction

When academic publications are retracted, retraction notices are usually issued. The decision to retract publications may involve multiple institutions and can be complex. According to the flowcharts proposed by the Committee on Publication Ethics (COPE) (COPE Council, 2020), as well as the two-stage retraction process recommended by the Editor-in-Chief of *Science* journal (Thorp, 2022), upon receiving credible allegations of research misconduct, journal authorities (i.e., editors and publishers) should contact authors for clarification, and only in cases of no author response or unsatisfactory author explanations is it advisable that journal authorities request research performing organizations (i.e., institutions that authors of retracted publications are affiliated with) to launch investigations^1^. Ideally, research performing organizations would readily accept journal authorities’ requests and conduct investigations promptly, transparently, and effectively. However, research performing organizations may disregard external requests for investigations or behave uncooperatively (Fiona, 2011; Marcus & Oransky, 2017; Xu & Hu, 2021). Journal authorities may sometimes have to make retraction decisions solely based on their own investigations (e.g., in cases of plagiarism or self-plagiarism of published works) or without any investigation at all (e.g., in cases of refusal to pay publication fees for accepted manuscripts published online ahead of print^2^) or based on hard evidence from whistle-blowers (e.g., in cases of plagiarism or image manipulation). Journal authorities may also retract publications when notified by peer journal authorities of confirmed duplicate publications. Research performing organizations and research funding organizations may conduct in-house investigations and communicate their findings to journal authorities, which may lead to the latter’s retraction decisions. It may also happen that journal authorities end up being unsatisfied with research performing organizations’ investigation findings, which may or may not lead to retraction. Self-retraction is another possibility, in which researchers detect problems with their own publications, sometimes prompted by a peer, self-report them to journal authorities, and request a retraction (Vuong, 2019). In practice, it takes various stakeholders’ joint efforts to retract a publication when indicted. Even when other stakeholders are collaborative, journal authorities may ignore or respond slowly to credible allegations raised by individual whistle-blowers (Grey et al., 2020) or even to requests for self-retractions due to honest error (Hosseini et al., 2018), and publishers may ignore or disagree with requests for retraction from their own journal editors (Bolland et al., 2022).

The importance of institutional handling of alleged breaches of research integrity and ethics is highlighted in various research integrity-promoting guidelines (e.g., All European Academies, 2017; European Network of Research Integrity Offices [ENRIO] & The European Network for Research Ethics and Integrity [ENERI], 2019) and empirical research on the topic (e.g., Mejlgaard et al., 2020). However, previous studies (e.g., Alfredo & Hart 2011; Franzen, 2021; Golden et al., 2023; Grey et al., 2019; Gunsalus et al., 2018; Harvey, 2020) have focused on research performing organizations’ investigations into alleged breaches of research integrity and ethics but neglected the role of other institutions, such as research funding organizations and journal authorities. Hesselmann and Reinhart’s (2021) analysis of 127 retraction notices focused on the representation of different actors in retraction notices, but did not look at how often retraction notices mentioned institutional actors’ investigations into allegations leading to retractions and what types of institutional actors investigated those allegations. Although COPE highlights a pivotal role that research performing organizations and journal authorities should play in handling various forms of allegations (COPE Council, 2020), the initial set of the COPE retraction guidelines (COPE Council, 2009) does not mention anything about disclosing in retraction notices institutional investigations into allegations leading to retraction. However, the COPE retraction guidelines updated ten years later suggests citing institutional investigations to substantiate retraction reasons disclosed in retraction notices (COPE Council, 2019). Given the growing calls for transparency in handling retractions (Retraction Watch, n.d.-a; Teixeira da Silva & Vuong, 2022; Vuong, 2020), there is reason to expect increasing disclosure of institutional investigations in retraction notices if such an investigation took place and its findings led to the retraction. However, this expectation needs to be verified with empirical data from institutional investigations and their eventual corresponding retraction notices. Moreover, disciplinary variations have been found in authorship of retraction notices (Xu & Hu, 2018), the generic structure of retraction notices (Xu & Hu, 2021), and types and severity of reasons for retraction (Xu & Hu, 2022b, c). Therefore, it would be interesting to find out whether disclosure of institutional investigations into allegations in retraction notices varies across disciplines as well. Given the research gaps identified above, this study set out to answer the following four research questions:

1. What types of institutions investigate allegations leading to retractions?
2. How often do retraction notices mention such investigations by each type of institutions?
3. Does this differ between retractions before and after the issuing of the initial set of COPE retraction guidelines?
4. Does this differ between biomedical and natural sciences on the one hand and the social sciences and the humanities on the other hand?

## Methods

### Data Collection and Classification

Following a four-step procedure, as detailed by Xu and Hu (2022b), a total of 7,650 retraction notices were collected, which were published before 2020 and indexed in the three main sub-databases of the Web of Science (WoS) Core Collection, namely Science Citation Index–Expanded (SCIE), Social Sciences Citation Index (SSCI), and Arts & Humanities Citation Index (A&HCI). This study was part of a larger project, most of whose data were collected before the Retraction Watch Database (http://retractiondatabase.org), which has become the most complete source of retractions, was made available online. Our research focus was on retractions from journals indexed by the WoS whereas the Retraction Watch Database does not provide information about whether its retraction data are indexed by the WoS. It should be noted that our collection of retraction notices did not take into consideration the document type of retracted publications. We excluded 332 retraction notices of Cochrane reviews which were out-dated or replaced by a new version (Xu & Hu, 2022c). As a result, the remaining 7,318 retraction notices published between 1927 and 2019 constituted the dataset for our study.

To assess institutional investigations over time, the retraction notices in the dataset were classified by retraction period for analysis. Publication year of retraction notices was adopted as retraction year, which was retrieved as meta-data of the WoS-indexed retraction notices collected^3^. Following Xu and Hu (2022c), we divided retraction years into two retraction periods, namely from 1927 to 2009 and from 2010 to 2019 (Table 1). This classification was made for two reasons. First, the COPE retraction guidelines were introduced in late November of 2009, and the Retraction Watch (https://retractionwatch.com) was launched in August 2010. These events are expected to have influenced retraction practices. Second, substantial efforts have been made since 2010 by both national and international organizations to promote research integrity, such as World Conferences on Research Integrity (https://wcrif.org), European Network of Research Integrity Offices (www.enrio.eu), and Association for the Promotion of Research Integrity (www.aprin.or.jp), which might have impacted on institutional investigations into allegations. The COPE retraction guidelines introduced in late 2009 appeared to widely influence the practice of handling retractions as they are often cited in retraction notices to justify retraction decisions (Xu & Hu, 2021, 2022a), and the influence may have been reinforced by the above-mentioned events occurring in 2010 and onwards. We acknowledge the existence of a possible “impact” lag, but it is not possible to ascertain the length of that lag and just 3.9% (*n* = 295) of the retraction notices in our dataset were published in 2010 and were possibly subject to prior practices.

To assess disciplinary differences in institutional investigations, all the retraction notices in the dataset were classified by the three main sub-databases of the WoS Core Collection. Specifically, all the retraction notices indexed only in the SCIE were categorized as biomedical and natural sciences, whereas all those indexed in the SSCI and/or A&HCI but not in the SCIE were categorized as social sciences and the humanities. All the retraction notices indexed in both the SCIE and the SSCI and/or A&HCI were excluded (Table 1).

**Table 1:** Distributions of the retraction notices by retraction period and scholarly discipline

Retraction period			Discipline field		
1927–2009	2010–2019		B&NSs	SS&Hs	SCIE + SSCI/A&HCI
1,080	6,238		6,838	315	165

*Notes.* B&NSs = biomedical and natural sciences; SS&Hs = social sciences and the humanities

### Data Coding and Analysis

Informed by the literature (COPE Council, 2020; ENRIO & ENERI, 2019; Grey et al., 2019; Sørensen et al., 2021; Xu & Hu, 2021) and the Retraction Watch Database, a tentative coding scheme was developed based on the first author’s pilot coding of 22% (*n* = 1,609) of the dataset. The tentative coding scheme identified five distinct types of institutional authorities whose investigations led to a retraction, namely journal authorities, research performing organizations, research funding organizations, research integrity or ethics governing bodies, and third-party institutions. The tentative coding scheme was then used to analyze all the remaining retraction notices in the dataset, which resulted in the identification of one additional type of institution: unspecified institutions. The expanded coding scheme was then refined through discussion among the research team members, and used for an inter-coder reliability test, which led to finalizing it with slight modifications. The modifications focused on how to deal with cases in which an institution played two roles at the same time (i.e., research performing organization and research funding organization) or was consulted by an investigating institution. The Supplementary Information to this paper details how the coding scheme was developed.

Ideally, the concept of investigation should be defined and operationalized clearly. It can mean that an ad hoc or standing committee conducted a formal investigation, that an expert opinion was requested, or that questions were asked to the authors or their institutions. However, due to the uninformativeness and opacity of retraction notices (Grey et al., 2022; Hesselmann & Reinhart, 2021; Teixeira da Silva & Vuong, 2022; Vuong, 2020), it was technically impossible to ascertain whether investigations satisfied pre-set criteria. Therefore, we adopted a linguistic approach to define and identify institutional investigations. Specifically, we looked at whether institutional investigations disclosed in the retraction notices were identified through an explicit identifier of institutions (e.g., proper names of institutions, nouns and pronouns referring to institutions), together with a lexical marker indicating investigation (i.e., *investigate*, *review*, *evaluate*, *assess*, *examine*, *inquiry*, and the variants of these six words) and/or suggesting confirmation or discovery of retraction-engendering problems with retracted publications (e.g., *determine*, *conclude*, *find*, *discover*, and *establish*). Illustrative examples and more detail are made available in the Supplementary Information.

To establish coding reliability, a subset of the data was coded independently by the first two authors. Before the inter-coding exercise, the second author was trained by the first author to identify different types of institutional investigations into allegations, using the revised coding scheme. After the training, the two authors independently coded 366 (5%) retraction notices randomly selected from the dataset. A Cohen’s kappa test indicated excellent inter-coder agreement (*k* = 0.912). Disagreements on coding were resolved through discussion between the first two authors. Subsequently, using the finalized coding scheme, the first author coded all the remaining data. Full details of our data coding are presented in the Supplementary Information.

Each retraction notice in the dataset was coded for investigations conducted by six groups of institutional stakeholders, namely journal authorities, research performing organizations, research funding organizations, research integrity and ethics governing bodies, third-party institutions, and unspecified institutions. If more than one of the six entities were described in the same retraction notice as being involved in an investigation, independently or collaboratively, they were categorized as joint institutions. As a result, only one of the seven types of institutional entities was coded dichotomously (i.e., present vs. absent) in all the retraction notices in which institutional investigations were disclosed. A series of two-way Chi-square (continuity correction) tests was carried out to assess the differences between the two retraction periods (i.e., disclosed vs. undisclosed x before vs. since 2010) and the two disciplinary groupings (i.e., disclosed vs. undisclosed x biomedical and natural sciences vs. social sciences and the humanities). When the expected frequencies in a cell of the contingency tables was less than 5, no Chi-square tests were run. The statistical analyses were conducted with Jamovi (The Jamovi project, 2021), a R-based software that generates Chi-square test statistics (2 × 2 contingency tables). Only Chi-square test results showing significant differences are reported in the Results section below.

## Results

In 26.3% (*n* = 1,925) of the 7,318 retraction notices examined, institutional investigations into allegations that had led to retractions were identified, whereas no institutional investigations could be identified in the remaining 73.7% (*n* = 5,393) of the dataset. As indicated in the third column of Table 2, institutional investigations conducted by journal authorities (JAs) or research performing organizations (RPOs) were disclosed in 22.4% of all the retraction notices. By contrast, the other five types of institutional entities combined, namely joint institutions (JIs), research integrity and ethics governing bodies (RIEGBs), third-party institutions (TPIs), unspecified institutions (UIs), research funding organizations (RFOs ), were mentioned in only 3.9% of the dataset. Table 2 also presents the frequency and prevalence of investigations conducted by the seven types of entities by retraction period and disciplinary field.

**Table 2:** Frequency and prevalence of institutional investigation by institution type, retraction period, and disciplinary field

Investigation conducted by	Full dataset (*N* = 7,318)		Retraction period (*N* = 7,318)				Disciplinary field (*N* = 7,153)			
			1927–2009 (*n* = 1,080)		2010–2019 (*n* = 6,238)		SS&Hs (*n* = 315)		B&NSs (*n* = 6,838)	
	*n*	*%*	*n*	%	*n*	%	*n*	%	*n*	%
JAs	884	12.1	52	4.8	832	13.3	45	14.3	819	12.0
RPOs	753	10.3	117	10.8	636	10.2	70	22.2	672	9.8
JIs	139	1.9	20	1.9	119	1.9	3	1.0	136	2.0
RIEGBs	76	1.0	10	0.9	66	1.1	0	0.0	75	1.1
TPIs	33	0.5	0	0.0	33	0.5	1	0.3	21	0.3
UIs	30	0.4	2	0.2	28	0.4	3	1.0	27	0.4
RFOs	10	0.1	5	0.5	5	0.1	0	0.0	10	0.1

*Notes.* SS&Hs = social sciences and the humanities; B&NSs = biomedical and natural sciences

The retraction notices published since 2010 were more likely than those published before 2010 to disclose investigations by journal authorities, χ^2^ (1, *N* = 7,318) = 62.2, *p* < .001. The retraction notices in social sciences and the humanities were more likely than those in biomedical and natural sciences to disclose investigations by research performing organizations, χ^2^ (1, *N* = 7,153) = 48.4, *p* < .001.

## Discussion

Before discussing our research findings, we should highlight that our study had its limitations. First, since this study drew on retraction notices as its only data source, it cannot answer any question about whether institutional investigations were conducted and should have been mentioned in the retraction notice, but rather whether such investigations were reported in retraction notices. As suggested by the COPE retraction guidelines (COPE Council, 2009, 2019), retraction notices may be consensual results of negotiations between journal authorities and authors of retracted publications. Indeed, retraction notices can be quite vague with a view to obtaining non-litigious acceptance by the authors of retracted publications. Consequently, the identified prevalence of disclosure of institutional investigations in retraction notices may have under-represented the *de facto* frequency of institutional investigations leading to a retraction. Second, our data source was restricted to the WoS and did not include useful information not indexed by that database. Future research should explore additional data sources, such as Retraction Watch database and blogs, investigation reports by institutional stakeholders, and surveys and interviews with institutional stakeholders and retraction notice authors. Third, the broad binary distinction between disciplines adopted in our study did not allow the detection of more nuanced disciplinary differences in retraction notices’ disclosure of investigations. Future studies can consider investigating more fine-grained classifications of academic disciplines to uncover potential discipline-specific trends. Fourth, our study examined only two contextual factors potentially influencing retraction notices’ disclosure of institutional investigations. Follow-up studies can examine whether, for instance, retraction notice authorship and severity of retraction reasons would also be influencing factors. Fifth, the study did not examine the content of the information about institutional investigations disclosed in retraction notices. Future research on what institutional investigations actually contributed to the decision-making process of retraction can deepen our understanding of the roles played by institutional stakeholders in the operation of the mechanism of retraction.

### Disclosure of Institutional Investigations

The identified seven distinct types of institutional investigators indicates that the decision-making process of retraction can involve both individual and collective engagement of various institutional stakeholders. The majority of the institutional investigations disclosed in the retraction notices were conducted by journal authorities and research performing organizations. The study also identified retraction notices’ infrequent reporting of institutional investigations, a pattern which might have arisen in part from the lack of guidance on reporting institutional investigations in the first set of COPE retraction guidelines (COPE Council, 2009). If, however, the lack of reporting actually reflects a lack of investigations, this might be due to multiple factors, for example, the high costs of institutional investigations (Gammon & Franzini, 2013; Michalek et al., 2010), inadequate awareness of the importance of handling allegations (Sørensen et al., 2021), prioritization of literature correction over post-retraction investigations (Thorp, 2022), and difficulties in handling allegations in general (Wager, 2007; Wager & Kleinert, 2021), particularly when allegations involve multiple research performing organizations (Hesselmann & Reinhart, 2021), especially if these are based in more than one country (Boesz & Lloyd, 2008). It should also be noted that institutional investigations may not necessarily always take place before retraction notices occurs. For example, as suggested by Thorp (2022), an institutional investigation may be initiated after the retraction of a publication or have been prompted by a journal notifying authors of a decision to retract (Marcus, 2016). Additionally, retractions and institutional investigations may occur simultaneously and with no clear indication as to who/what prompted the investigations, and such timing would prevent retraction notices from mentioning institutional investigations. We believe that retraction notices need to explain why a retraction took place. If an investigation prompted it, this needs to be mentioned, including the fact that the report of the investigation has not been made public if that is the case. We consider mentioning simultaneous or later investigations optional. Most journals will make their own assessment or even do a full investigation before retracting an article. We recommend that this should be mentioned in the retraction notice as it documents that the journal cares about quality assurance.

It is, however, expected that only in rare cases, such as refusal to pay publication fees for accepted manuscripts published online ahead of print (Xu & Hu, 2022b) and administrative errors by journal authorities (Xu & Hu, 2022c), retraction decisions can be made without any institutional investigation. Therefore, the identified infrequent disclosure of institutional investigations is likely to under-represent the actual prevalence of institutional investigations and suggests a worrying lack of transparency about institutional investigations in retraction notices. This interpretation is consistent with Hesselmann and colleagues’ (2017) observation that retraction notices are “obscuring the actors and processes through which retractions are effected” (p. 816). One explanation for this can be that “processes [of investigating scientific misconduct] often are considered highly confidential” (Hesselmann & Reinhart, 2021, p. 428), and confidentiality and anonymity protections may be used as a cover for research misconduct (Dougherty, 2021). Institutional investigations may not have been mentioned to avoid disclosing stigmatizing information, such as identities of misbehaving researchers and disciplinary actions taken against them (Xu & Hu, 2022a) or to prevent possible litigations from authors of retracted publications (COPE Council, 2009, 2019; Wager & Williams 2011). Authorship of retraction notices can be another explanation for the obscuration of institutional investigations^4^. Retraction notices are mainly issued by journal authorities and/or authors of retracted publications (Xu & Hu, 2018, 2022a). By keeping silent about institutional investigations into allegations against themselves, authors of retracted publications not only avoid disclosing sensitive details about their misbehaviors, but also create an impression of self-retraction. Since journal authorities can justify retraction decisions by citing the COPE retraction guidelines and journal policies on retraction (Xu & Hu, 2021), they may have found it unnecessary to mention institutional investigations as a further justification for their retraction decisions.

Despite the above possible reasons for the observed infrequent disclosure of institutional investigations, we argue for increasing transparency about the decision-making process of retraction by disclosing institutional investigations in retraction notices more frequently. In cases where institutional investigations take place when retraction decisions are being made or after retraction notices are issued, published retraction notices should be updated to mention institutional investigations. Retraction notices always can and should explicitly mention whether institutional investigations have been conducted and by whom, even in cases where no misconduct is found or where there are local policies on privacy protection and disclosure of sensitive information. Disclosure of institutional investigations can justify retraction decisions and thus help protect journal authorities from litigations from misbehaving researchers (COPE Council, 2019; Wager & Kleinert 2021). More importantly, it sends a clear message that various institutional stakeholders are doing their job, either independently or collaboratively, to uphold the integrity of research and publication norms. Such a message can deter potential offence and win back the public’s trust in the self-governance and self-correction of academic research. Frequent disclosure of post-publication institutional investigations into allegations can mitigate the damage to the reputation of journal authorities and research performing organizations caused by their failure to safeguard the integrity of research and publications in the first place (Xu & Hu, 2022d). Since the research community tends to show low support for editors’ handling of research misconduct (Hesselmann & Reinhart, 2021), it is in the interest of journal authorities to publicize their investigations into allegations more frequently via retraction notices, because doing so can help promote their image as competent and trustworthy gatekeepers of research integrity and ethics. We would like to emphasize that retraction notices should disclose institutional investigations as frequently as possible to ensure retraction transparency and enjoy the benefits discussed above. Given the first limitation of our study mentioned earlier, we do not know how many retraction notices should have mentioned institutional investigations. It is hoped that future research can answer such an important question.

### Time Trends and Disciplinary Differences

The retraction notices published after the COPE guidelines on retraction became available were more likely to disclose investigations by journal authorities. Since their introduction around 2010 (Retraction Watch, n.d.-b; Xu & Hu, 2022c), third parties like web-based platforms of post-publication peer review (e.g., Retraction Watch and PubPeer) have tended to openly expose serious problems with publications and underlying scientific misconduct, and the evidence made available publicly, once confirmed, could be readily recognized and cited by journal authorities to justify their retraction decisions (Bik, 2019; Brookes, 2014). The COPE flowcharts highlight a pivotal role that research performing organizations should take in handling a variety of misconduct allegations (COPE Council, 2020). However, research performing organizations may have been unable to investigate allegations against their employees effectively due to a lack of guidelines (National Academies of Sciences, 2017; National Academies of Sciences et al., 2016), or disinclined to do so to prevent negative consequences of eventual retractions, such as reputational damage and financial loss (Institute of Medicine, 2012), leaving their obligation to investigate allegations to be fulfilled by journal authorities. Research performing organizations may have conducted investigations into allegations against their misbehaving employees but subsequently have decided to cover them up (Nouchi et al., 2020) by soft-pedalling the severity of confirmed allegations (e.g., Tsukumo et al., 2016), producing research misconduct reports with inadequate credibility (Gunsalus et al., 2018), or refusing to reveal their investigations (Abdi et al., 2023). As a result, journal authorities may have ended up making retraction decisions based on their own investigations (e.g., The Journal of Rheumatology, 2018) to ward off litigations, which can be a plausible explanation for the observed increase in disclosing their investigations into allegations.

The retraction notices in social sciences and the humanities were more likely than those in biomedical and natural sciences to disclose investigations by research performing organizations. This trend might have to do with the types of reasons for retraction that prevailed in the two disciplinary groupings. Certain types of retraction reasons (e.g., image and data manipulation) that are common in biomedical and natural sciences can be more readily identified in the publications, for instance, by third-party academic sleuths (Bik, 2019; Brookes, 2014). Therefore, it could be less necessary for journal authorities to make retraction decisions based on investigations by research performing organizations.

## Conclusion

Our study identified seven types of institutional investigators into allegations that had led to retractions, and institutional investigations were disclosed in just over one quarter of the 7,318 retraction notices examined. If this finding under-represents *de facto* institutional investigations, there is a need to disclose institutional investigations in retraction notices more frequently. If the identified infrequent disclosure of institutional investigations reflected *de facto* institutional investigations, there is an even more urgent need for institutional stakeholders to investigate allegations about any retraction-engendering behaviors (e.g., misconduct, questionable research practices, and honest error) more frequently and make their work publicly known through retraction notices. In either case, the finding points to a substantial lack of transparency about the decision-making process of retraction. Our study also identified two contextual factors, namely retraction period (i.e., 1927–2009 vs. 2010–2019) and disciplinary field (i.e., biomedical and natural sciences vs. social sciences and the humanities), which were associated with retraction notices’ disclosure of investigations. This finding reveals the context-specificity of retraction notices’ disclosure of institutional investigations into allegations. While the first set of COPE retraction guidelines (COPE Council, 2009) did not require disclosure of institutional investigations in retraction notices, the latest set of COPE retraction guidelines (COPE Council, 2019) just makes it optional to disclose institutional investigations into allegations, despite the various benefits of doing so, as discussed earlier. Therefore, we suggest that the COPE retraction guidelines in the future move forward to make such disclosure mandatory. To encourage this, journal authorities, research funding organizations, and online watchdogs of research integrity and ethics should reward research performing organizations with due recognition for investigating allegations and disclosing in retraction notices confirmed allegations.

## Electronic supplementary material

Below is the link to the electronic supplementary material.

Supplementary Material 1

## Footnotes

1 In cases of well-founded suspicions or evidence of data falsification or fabrication, journals should consider contacting the research performing organizations at the same time as, or before, reaching out to the author(s) to prevent the latter’s destruction or alteration of evidence (Wager & Kleinert, 2021).

2 Xu and Hu (2022) and the retraction reason field of the Retraction Watch Database.

3 It should be noted that one reviewer of this article pointed out that some publishers do not mention the date of retraction on the retracted publication. We did not look into this as we took retraction date as the publication date of retraction notices as indicated on WoS.

4 While journal authorities are believed to have the final say on what to disclose in retraction notices, it is undeniable that authors of retracted publications may have some influence on whether to reveal institutional investigations.

## References

[CR1] Abdi, S., Nemery, B., & Dierickx, K. (2023). What criteria are used in the investigation of alleged cases of research misconduct? *Accountability in Research*, *30*(2), 109–131. 10.1080/08989621.2021.1973894. 10.1080/08989621.2021.1973894 34455868

[CR38] Abou-Raya, A., Abou-Raya, S., Khadrawi, T., & Helmii, M. (2018). RETRACTED ARTICLE: Effect of low-dose oral prednisolone on symptoms and systemic inflammation in older adults with moderate to severe knee osteoarthritis: A randomized placebo-controlled trial. *The Journal of Rheumatology*, *45*(12), 1713. 10.3899/jrheum.130199.RET1. 10.3899/jrheum.130199 24293578

[CR2] Alfredo K, Hart H (2011). The university and the responsible conduct of research: Who is responsible for what?. Science and Engineering Ethics.

[CR3] All European Academies (2017). *The European code of conduct for research integrity*. https://www.allea.org/wp-content/uploads/2017/05/ALLEA-European-Code-of-Conduct-for-Research-Integrity-2017.pdf

[CR4] Bik, E. (2019, July 16). PubPeer – a website to comment on scientific papers. Science Integrity Digest. https://scienceintegritydigest.com/2019/07/16/pubpeer-a-website-to-comment-on-scientific-papers/

[CR5] Boesz C, Lloyd N (2008). Investigating international misconduct. Nature.

[CR6] Bolland, M., Avenell, A., & Grey, A. (2022, November 4). How many ducks do you need to line up to get a publication retracted? Retraction Watch. https://retractionwatch.com/2022/11/04/how-many-ducks-do-you-need-to-line-up-to-get-a-publication-retracted/

[CR7] Brookes PS (2014). Internet publicity of data problems in the bioscience literature correlates with enhanced corrective action. PeerJ.

[CR8] Committee on Publication Ethics Council (2009). *30**November 2009:**Retraction guidelines*. Retrieved December 20, 2022 from https://publicationethics.org/newsevents/cope%E2%80%99s-retraction-guidelines.

[CR9] Committee on Publication Ethics Council (2019). *10 December 2019*: *Retraction guidelines*. Retrieved December 20, 2022 from https://publicationethics.org/retraction-guidelines

[CR10] Committee on Publication Ethics Council (2020). *Flowcharts*. Retrieved March 1, 2021 from https://publicationethics.org/guidance/Flowcharts

[CR11] Dougherty MV (2021). The use of confidentiality and anonymity protections as a cover for fraudulent fieldwork data. Research Ethics Review.

[CR12] European Network of Research Integrity Offices, & The European Network of Research Ethics and Research Integrity (2019). Recommendations for the investigation of research misconduct: ENRIO handbook. Jahrbuch für Wissenschaft und Ethik.

[CR13] Fiona G (2011). Institutional research misconduct: Failings over the MMR scare may need parliamentary inquiry. British Medical Journal.

[CR14] Franzen, S. (2021). *University responsibility for the adjudication of research misconduct: The science bubble* (1 ed.). Springer.

[CR15] Gammon E, Franzini L (2013). Research misconduct oversight: Defining case costs. Journal of Health Care Finance.

[CR16] Golden J, Mazzotta CM, Zittel-Barr K (2023). Systemic obstacles to addressing Research Misconduct in Higher Education: A Case Study. Journal of Academic Ethics.

[CR17] Grey, A., Avenell, A., & Bolland, M. (2022). Timeliness and content of retraction notices for publications by a single research group. *Accountability in Research*, *29*(6), 347–378. 10.1080/08989621.2021.1920409. 10.1080/08989621.2021.1920409 33882262

[CR18] Grey A, Avenell A, Gamble G, Bolland M (2020). Assessing and raising concerns about duplicate publication, authorship transgressions and data errors in a body of preclinical research. Science and Engineering Ethics.

[CR19] Grey A, Bolland M, Gamble G, Avenell A (2019). Quality of reports of investigations of research integrity by academic institutions. Research Integrity and Peer Review.

[CR20] Gunsalus CK, Marcus AR, Oransky I (2018). Institutional research misconduct reports need more credibility. Journal Of The American Medical Association.

[CR21] Harvey L (2020). Research fraud: A long-term problem exacerbated by the clamour for research grants. Quality in Higher Education.

[CR22] Hesselmann F, Graf V, Schmidt M, Reinhart M (2017). The visibility of scientific misconduct: A review of the literature on retracted journal articles. Current Sociology.

[CR23] Hesselmann, F., & Reinhart, M. (2021). Cycles of invisibility: The limits of transparency in dealing with scientific misconduct. *Social Studies of Science*, *51*(3), 414–438. 10.1177/0306312720975201. 10.1177/0306312720975201 33234058

[CR24] Hosseini M, Hilhorst M, de Beaufort I, Fanelli D (2018). Doing the right thing: A qualitative investigation of retractions due to unintentional error. Science and Engineering Ethics.

[CR25] Institute of Medicine. (2012). *Evolution of translational omics lessons learned and the path forward*. National Academies Press. 24872966

[CR26] Marcus, A. (2016). Neuro journal pulls article for data theft, prompts misconduct probe. Retraction Watch, May 31, 2016. https://retractionwatch.com/2016/05/31/neuro-journal-pulls-article-for-data-theft-prompts-misconduct-probe/

[CR27] Marcus, A., & Oransky, I. (2017). Is there a retraction problem? And, if so, what can we do about it? In K. H. Jamieson, D. Kahan, & G. Scambler (Eds.), *The Oxford handbook of the science of science communication* (pp. 119–126). Oxford University Press. 10.1093/OXFORDHB/9780190497620.013.13

[CR28] Mejlgaard N, Bouter LM, Gaskell G, Kavouras P, Allum N, Bendtsen AK, Charitidis CA, Claesen N, Dierickx K, Domaradzka A, Elizondo AR, Foeger N, Hiney M, Kaltenbrunner W, Labib K, Marušić A, Sørensen MP, Ravn T, Ščepanović R, Veltri GA (2020). Research integrity: Nine ways to move from talk to walk. Nature.

[CR29] Michalek, A. M., Hutson, A. D., Wicher, C. P., & Trump, D. L. (2010). The costs and underappreciated consequences of research misconduct: A case study. *PLoS Medicine*, *7*(8), Article e1000318. 10.1371/journal.pmed.1000318. 10.1371/journal.pmed.1000318 PMC2923086 20808955

[CR30] National Academies of Sciences, Medicine and Engineering (2017). *Addressing research misconduct and detrimental research practices: Current knowledge and issues* (pp. 105–146). National Academies Press.

[CR31] National Academies of Sciences, Medicine and Engineering, Policy and Global Affairs, Board on Higher Education and Workforce, Committee on Science, Technology and Law. Committee on Federal Research Regulations and Reporting Requirements: A new Framework for Research Universities in the 21 Century. *Optimizing the nation’s investment in academic research: A new regulatory framework for the 21st century*. National Academies Press. 10.17226/21824

[CR32] Nouchi R, Aihara H, Arie F, Asashima M, Daida H, Fudano J, Fujiwara Y, Fushiki S, Geller RJ, Hatano K, Homma T, Kimura M, Kuroki T, Miki K, Morita I, Nitta K, Shinohara A, Siomi MC, Yoshida M, Ichikawa I (2020). Toward global standardization of conducting fair investigations of allegations of research misconduct. Accountability in Research.

[CR33] Retraction Watch. (n.d.-a). The Retraction Watch Transparency Index. Retraction Watch. https://retractionwatch.com/the-retraction-watch-faq/transparencyindex/

[CR34] Retraction Watch. (n.d.-b). What people are saying about Retraction Watch. Retraction Watch. https://retractionwatch.com/what-people-are-saying-about-retraction-watch/

[CR35] Sørensen MP, Ravn T, Marušić A, Elizondo AR, Kavouras P, Tijdink JK, Bendtsen AK (2021). Strengthening research integrity: Which topic areas should organisations focus on?. Humanities and Social Sciences Communications.

[CR36] Teixeira da Silva JA, Vuong QH (2022). Fortification of retraction notices to improve their transparency and usefulness. Learned Publishing.

[CR37] The Jamovi Project (2021). *Jamovi*. In (Version 2.2) https://www.jamovi.org

[CR39] Thorp HH (2022). Rethinking the retraction process. Science.

[CR40] Tsukumo, D. M., Carvalho-Filho, M. A., Carvalheira, J. B., Prada, P. O., Hirabara, S. M., Schenka, A. A., Araujo, E. P., Vassallo, J., Curi, R., Velloso, L. A., & Saad, M. J. (2016). Statement of retraction. Loss-of-function mutation in toll-like receptor 4 prevents diet-induced obesity and insulin resistance. *Diabetes* 2007;56:1986–1998. 10.2337/db06-1595; Statement of Retraction, *Diabetes*, *65*(4), 1126–1127. 10.2337/db16-rt04a. 10.2337/db16-rt04a 27208024

[CR41] Vuong QH (2019). The limitations of retraction notices and the heroic acts of authors who correct the scholarly record: An analysis of retractions of papers published from 1975 to 2019. Learned Publishing.

[CR42] Vuong QH (2020). Reform retractions to make them more transparent. Nature.

[CR43] Wager E (2007). What do journal editors do when they suspect research misconduct?. Medicine and Law.

[CR44] Wager, E., & Kleinert, S. (2021). Cooperation & liaison between universities & editors (CLUE): Recommendations on best practice. *Research Integrity and Peer Review*, *6*(1), Article 6. 10.1186/s41073-021-00109-3. 10.1186/s41073-021-00109-3 PMC8048029 33853690

[CR45] Wager E, Williams P (2011). Why and how do journals retract articles? An analysis of Medline retractions 1988–2008. Journal of Medical Ethics.

[CR46] Xu, S. B., & Hu, G. (2018). Retraction notices: Who authored them? *Publications*, *6*(1), 10.3390/publications6010002.

[CR47] Xu, S. B., & Hu, G. (2021). Retraction notices as a high-stakes academic genre: A move analysis. In K. L. Lin, I. N. Mwinlaaru, & D. Tay (Eds.), *Approaches to specialized genres* (pp. 101–120). Routledge. 10.4324/9780429053351

[CR48] Xu SB, Hu G (2022). Construction and management of retraction stigma in retraction notices: An authorship-based investigation. Current Psychology.

[CR49] Xu SB, Hu G (2022). A cross-disciplinary and severity-based study of author-related reasons for retraction. Accountability in Research.

[CR50] Xu SB, Hu G (2022). Non-author entities accountable for retractions: A diachronic and cross-disciplinary exploration of reasons for retraction. Learned Publishing.

[CR51] Xu SB, Hu G (2022). Retraction stigma and its communication via retraction notices. Minerva.

