# Supplementary material for: What do Retraction Notices Reveal About Institutional Investigations into Allegations Underlying Retractions?
Source: Sci Eng Ethics. 2023 Jul 4;29(4):25. doi: 10.1007/s11948-023-00442-4 (PMC10319669; doi:10.1007/s11948-023-00442-4)
Supplement: Supplementary file 1 — Supplementary Material 1 [file 11948_2023_442_MOESM1_ESM.docx]

**Supplementary Information**

This digital supplement consists of four sections. It starts with defining institutional investigation, followed by an introduction to the scope of data coding. The third section presents the coding scheme developed for the study, and the last section details how the data were coded, using the coding scheme.

**Defining institutional investigation**

Institutional investigations disclosed in the retraction notices was identified through an explicit identifier of institutions (e.g., proper names of institutions and nouns and pronouns referring to institutions), together with a lexical marker ^[[1]](#footnote-1)^ indicating investigation (i.e., *investigate*, *review*, *evaluate*, *assess*, *examine*, *inquiry*, and the variants of these six words) and/or suggesting confirmation or discovery of retraction-engendering problems with retracted publications (e.g., *determine, conclude, find, discover,* and *establish*), as illustrated by Examples 1–3. Additionally, when a retraction decision followed a recommendation or request from, or authors’ admission to, a specific identifiable institution (or its individual representatives), an institutional investigation could be convincingly inferred and thus was coded as such even though none of the adopted lexical markers appeared in the retraction notice, as illustrated by Examples 4 and 5. When an institution was only mentioned as having been consulted during an investigation or informed of investigation findings, it was not considered as an institutional investigation by the institution, as illustrated by Example 6.

1. This article has been retracted by the editor because an investigation by the National Institutes of Health concluded that the data represented by Figures 3a and 4 were falsified. (RN-SCIE-0049)
2. Upon our follow up with the authors of the article, it was established that images for sample 35S::ZmARF25 plant #4 panel 4B (siliques) and 4C (plants) were generated by Photoshop software manipulation of images from other plants. (RN-SCIE-1502)
3. The publisher has discovered that the author(s) created and provided false information for the peer-review process. (RA-SCIE-0014-RN)
4. This article has been retracted at the request of the Department of Clinical Neuroscience, Karolinska Institute, Stockholm, Sweden. (RN-SCIE-2138)
5. The first author, Brandi M. Baughman, has admitted to the co-authors and the Office of Research Integrity at NIH that she falsified and/or fabricated data and text …. (RN-SCIE-0390)
6. The [US] Office of Research Integrity has been involved with the Emory University Investigation Committee and has been made aware of the Committee’s findings. (RN-SCIE-5157)

**Scope of data coding**

Our identification of institutional investigations into allegations drew solely on information disclosed in the retraction notices examined, and no other data sources were used except in two scenarios. First, when an institution could not be categorized unambiguously based on the information disclosed in a retraction notice, the corresponding retracted publication was searched for information to categorize it into one of five types of institutions, namely journal authorities, research performing organizations, research funding organizations, research integrity and ethics governing bodies, and third-party institutions. If in vain, the institution was then coded as an unspecified one. For instance, in Example 7, the National Institutes of Health is not listed as a research performing organization in the retraction notice but an author affiliation in the corresponding retracted publication, it was coded as a case of investigation by a research performing organization. When an institution (like the NIH in Example 5) was listed as both an author affiliation and a research funding organization, it was coded as investigation by a research preformation organization because the former rather than the latter was directly responsible for overseeing research activities and entitled to access raw data of alleged research. Second, in cases of ambiguous categorization of institutions, other retraction notices in the dataset were searched for information to confirm institution categories. In Example 8, the website does not tell which type of institution conducted an investigation, but the confusion was resolved through information disclosed in another retraction notice in the dataset (i.e., Example 9). The two exceptions to the use of only retraction notices under examination were grounded on the assumption that retraction notice writers did not intend to obscure institution-specifying information but avoided repeating the same information, especially in cases of handling multiple retractions at one time, as evidenced by Examples 8 and 9.

1. This article has been retracted by the editor because an investigation by the National Institutes of Health concluded that the data represented by Figures 3a and 4 were falsified. (RN-SCIE-0049)
2. A website (https://www.commissielevelt.nl/) contains a first set of publications by … that are now officially identified as fraudulent. (RN-SSCI-0328)
3. Our decision to issue this retraction was based on the October 31, 2011, report and recommendation from Tilburg University’s Levelt committee …. (RN-SSCI-0398)

**Coding scheme**

Informed by the literature and the Retraction Watch Database, a tentative coding scheme was developed based on the first author’s pilot coding of 22% (n = 1,609) of the dataset. The tentative coding scheme identified five distinct types of institutional authorities whose investigations led to a retraction, namely journal authorities, research performing organizations, research funding organizations, research integrity and ethics governing bodies, and third-party institutions. The tentative coding scheme was then used to analyze all the remaining retraction notices in the dataset, which resulted in identification of one additional type of institution: unspecified institutions. The expanded coding scheme was then refined through discussion among the research team members, and used for an inter-coder reliability test, which led to finalizing it with slight modifications. The modifications focused on how to deal with cases in which an institution played two roles at the same time (i.e., research performing organization and research funding organization) or was consulted by an investigating institution. The finalized coding scheme is as follows.

- *Investigation by journal authorities:* Journal editors and/or publishers launched and conducted investigations into retraction-engendering allegations, which may or may not involve invited individual external experts (e.g., individual experts, invited peer reviewers, statisticians, and editorial members of other journals).

1. The editors were made aware of concerns regarding potential manipulation of data in the article. An internal review by the editors determined that multiple Gapdh bands appear to be duplicated in Fig. 3A …. (RN-SCIE-9839)
2. Following an investigation, the Executive Editor has established that the acceptance of this article was based on at least one fake reviewer report. (RN-SCIE-0197)

- *Investigation by research performing organizations:* Research performing organizations conducted investigations into retraction-engendering allegations on their own initiative or as requested by journal authorities.

1. The retraction has been agreed following an investigation carried out by the National University of Singapore due to major overlap with a previously published article …. (RN-SSCI-0175)
2. Pfizer has undertaken a review and determined that there are duplications in Fig 4A of the article and that part of the experiments cannot be verified. (RN-SCIE-0206)

- *Investigation by research funding organizations:* Research funding organizations conducted investigations into retraction-engendering allegations on their own initiative or as requested by journal authorities.

1. Following the notice of Retraction and Replacement, the funder of this study informed us [authors of the retracted publication] of another important error. (RN-SCIE-0047)
2. The Journal was informed by the organization that sponsored the study, Boston Scientific, that errors were made during their own (i.e. Boston Scientific) data compilation …. (RN-SCIE-0094)

- *Investigation by research integrity and ethics governing bodies:* Research integrity and ethics governing bodies at different levels beyond research performing organizations (e.g., governmental agencies and academic societies) investigated retraction-engendering allegations on their own initiative or as requested by research performing organizations, research funding organizations, or journal authorities.

1. An investigation by the Office of Research Integrity determined that falsified and/or fabricated Western blots were included in Figs. 1B, 3A, 4D, 5E, and 6C (https://ori.hhs.gov/content/case-summary-pastorino-john-g). (RN-SCIE-1444)
2. … the article … was found to involve blameworthy inaccuracies in the way the research was carried out …. The EUR Inquiry Committee on Scientific Integrity (CWI) commissioned by the Board of Erasmus University Rotterdam (EUR) has made this determination. (RN-SSCI-0251)

- *Investigation by third parties:* Institutions other than journal authorities, research performing organizations, research funding organizations, and research integrity and ethics governing bodies conducted investigations that led to retractions.

1. Moreover, we [the authors of the retracted publication] asked a third party to carry out independent NMR analysis and they determined that the MicroCombiChem compound had a nitrogen in the vicinity of a methyl group, which is the case for gentamicin B1 but not for G418. (RN-SCIE-9847)

- *Unspecified institutional investigation:* An institutional investigation is disclosed in retraction notices without specifying the institution that conducted them.

1. Following an internal investigation, the article referenced above has been found to contain inappropriate manipulation of bands in gels within Figures 2A, 3A, and 4. (RN-SCIE-2176)
2. After a thorough institutional investigation, it was concluded that there was incontrovertible evidence of improper manipulation of figures. (RN-SCIE-3874)
3. A local investigation was instigated and we received further correspondence dated 6 November 2008 including the following paragraph …. (RN-SCIE-5748)

**Data coding**

Using the data scheme above, each retraction notice in the dataset was coded for investigations conducted by six groups of institutional stakeholders, namely journal authorities, research performing organizations, research funding organizations, research integrity and ethics governing bodies, third-party institutions, and unspecified institutions. If more than one of the six institutional entities were described in the same retraction notice as being involved in an investigation, independently or collaboratively, they were categorized as joint institutions. As a result, only one of the seven types of institutional entities was coded dichotomously (i.e., present vs. absent) in all the retraction notices in which institutional investigations were disclosed.

- *Joint investigation*: At least two types of the above institutions investigated retraction-engendering allegations jointly or independently.

1. An Office of Research Integrity (ORI) report states that an investigation conducted by the University of Washington, where the original research was performed, and additional analysis conducted by the ORI found evidence that … [name of one of the co-authors of the retracted publication] engaged in research misconduct. (RN-SCIE-2848)

Notably, academic web-based platforms, like PubPeer and Retraction Watch in Examples 23 and 24, were identified as independent third-party institutions because they treat themselves as a collective and conduct investigations by putting retraction-engendering information from different sources. Moreover, in a few cases when a Research Integrity Office was mentioned in a retraction notice without specifying its institutional level, it was coded as the U.S. one, a national governing body of research integrity and ethics because it is well known as such. It should also be noted that institutional investigations which were pending, on-going, inconclusive, or unconfirmed, like in Examples 25 and 26, were excluded for analysis because our study focused on retraction-engendering institutional investigations.

1. As noted on PubPeer, Figures (a) and (b) show signs of figure duplication in the Western Blots …. We were unable to contact the authors. The Editorial Board recommended retracting the article. (RN-SCIE-12925)
2. We wish to retract the paper entitled … as a result of concerns about data integrity and scientific misconduct, which have been brought to our attention. For further information, see also https://retractionwatch.com/2016/11/09/analysis-casts-doubt-on-bone-researchers-body-of-work. (RN-SCIE-10858) (Note: The retraction notice was issued after the Retraction Watch post appeared.)
3. The University of Zabol are currently undertaking an investigation into the conduct of Dr Abkhoo. (RN-SCIE-1261)
4. The editor has been unable to confirm with Northwest University whether an institutional investigation has taken place. (RN-SCIE-0469)

1. The lexical markers were not pre-set but identified through a pilot manual coding of the whole dataset. Although journal authorities are believed to have made retraction decisions based on their own investigations in cases of lack of retraction-engendering investigations by other institutions, this criterion for identifying institutional investigations was adopted to uncover to what extent journal authorities obscured, intentionally or unintentionally, their involvement in the retraction decision-making process. [↑](#footnote-ref-1)
